# Preoperative magnetic resonance imaging use and oncologic outcomes in premenopausal breast cancer patients

**DOI:** 10.1038/s41523-020-00192-7

**Published:** 2020-10-02

**Authors:** Zexian Zeng, Amanda Amin, Ankita Roy, Natalie E. Pulliam, Lindsey C. Karavites, Sasa Espino, Irene Helenowski, Xiaoyu Li, Yuan Luo, Seema A. Khan

**Affiliations:** 1 Department of Preventive Medicine, Northwestern University Feinberg School of Medicine, Chicago, IL USA; 2 Department of Data Sciences, Dana-Farber Cancer Institute, Harvard T.H.Chan School of Public Health, Boston, MA USA; 3 Department of Surgery, Kansas University Medical Center, Kansas City, KS USA; 4 Department of Surgery, Northwestern University Feinberg School of Medicine, Chicago, IL USA; 5 Department of Surgery, University of Illinois College of Medicine at Mt. Sinai Hospital, Chicago, IL USA; 6 Tsinghua University, Beijing, China

**Keywords:** Breast cancer, Outcomes research

## Abstract

Breast magnetic resonance imaging (MRI) delineates disease extent sensitively in newly diagnosed breast cancer patients, but improved cancer outcomes are uncertain. Young women, for whom mammography is less sensitive, are expected to benefit from MRI-based resection. We identified 512 women aged ≤50 years, undergoing breast-conserving treatment (BCT: tumor-free resection margins and radiotherapy) during 2006–2013 through Northwestern Medicine database queries; 64.5% received preoperative MRI and 35.5% did not. Tumor and treatment parameters were similar between groups. We estimated the adjusted hazard ratios (aHR) for local and distant recurrences (LR and DR), using multivariable regression models, accounting for important therapeutic and prognostic parameters. LR rate with MRI use was 7.9 vs. 8.2% without MRI, aHR = 1.03 (95% CI 0.53–1.99). DR rate was 6.4 vs. 6.6%, aHR = 0.89 (95% CI 0.43–1.84). In 119 women aged ≤40, results were similar to LR aHR = 1.82 (95% CI 0.43–7.76) and DR aHR = 0.93 (95% CI 0.26–3.34). Sensitivity analyses showed similar results. The use of preoperative MRI in women aged ≤50 years should be reconsidered until there is proof of benefit.

## Introduction

A key aspect of local therapy of breast cancer is the complete resection of the breast tumor, with pathologically tumor-free margins. Imaging of the breast is a necessary component of the initial evaluation of the extent of disease in the breast and enables assessment of the feasibility of breast-conserving resection; i.e., imaging selects out women who are not candidates for breast conservation based on the extent of in-breast disease. The notion that more sensitive imaging will lead to better selection of surgical therapy to enable more complete surgical excision, and therefore improved local control is implicit in the care devoted to preoperative “extent of disease” evaluation. Currently, the most sensitive imaging modality for delineating the tumor extent is magnetic resonance imaging (MRI)^1–4^, yet its role in the preoperative evaluation of tumor extent remains controversial^2,5–8^. In multiple studies, the expected benefits of lower re-excision rates and improved short and long-term cancer outcomes have failed to materialize^9–11^, while the odds of receiving therapeutic mastectomy and contralateral prophylactic mastectomy are increased^9,10,12,13^. In particular, a meta-analysis of four studies (three retrospective and one prospective) does not show a clear benefit from preoperative breast MRI^6^. However, the published retrospective studies are of varying size, and most report few recurrence events^9,14–18^. Furthermore, there are differences in the follow-up time between the MRI and no-MRI groups in these studies, since women diagnosed later in the study period received MRI more frequently^15,17,19,20^. The available literature also suggests a propensity for more frequent MRI use in younger women^6,11,17,18,21–23^, which is likely driven by their greater breast density, their higher local recurrence risk, and the higher likelihood of genetic susceptibility. Thus, if MRI-based planning of surgical resection were to reduce cancer recurrence risk, it would be more likely to do so in younger women, but no study so far has specifically examined the effect of young age on cancer outcomes with and without MRI use.

In an attempt to clarify the potential benefit of preoperative breast MRI on long-term breast cancer outcomes in younger women (age ≤ 50 years), we constructed a retrospective cohort of 571 women diagnosed with primary breast cancer seen at the Lynn Sage Breast Center who underwent breast-conserving surgery performed at Northwestern Medicine. By excluding women diagnosed prior to 2006, we achieved a roughly balanced population of patients with and without preoperative MRI, with similar follow-up time and balanced tumor characteristics. In analyses of cancer outcomes in this young population, we tested the hypothesis that patients selected for BCS based on MRI evaluation experience better local control than those who were selected based on conventional imaging. We also report results of sensitivity analyses that excluded women with a follow-up time of <3 years and those diagnosed with DCIS.

## Results

### Cohort identification and data development

Women presenting to the Lynn Sage Breast Center of Northwestern Medicine with a diagnosis of primary Stage 0-III breast cancer between January 2006 and December 2013, who underwent breast-conserving surgery as the initial treatment were included in this study. Those receiving neoadjuvant therapy were excluded, as were 59 women for whom the use of postoperative radiotherapy could not be ascertained. Ethical approval for this study was obtained from the Northwestern University Institutional Review Board (IRB number STU00200923-MOD0006). Women with breast MRI performed within 60 days of primary breast cancer surgery were categorized as the MRI group and all others as the no-MRI group. Data on demographic, tumor-related, therapy-related, and outcome parameters were retrieved from the Northwestern Medicine Enterprise Data Warehouse^24^ and developed from free text using an in-house natural language processing (NLP) system^25–27^. These parameters are shown in Table 1. To obtain the ground truth for model development, information on a larger set of 937 women unrestricted by age was double annotated by two annotators. The inter-rater agreement for the two annotators (Cohen’s kappa score) was 0.92 for local recurrence and 0.87 for distant recurrence, both considered as excellent agreement^28^. Discordant items were resolved by consensus which included the senior author. The trained NLP systems were applied to 1108 non-annotated samples; their predicted recurrence status was then confirmed with manual chart review. These NLP systems have been validated and published in previous studies^25–27^. After the rigorous model development and chart review, a gold-standard dataset with validated information on 2045 women was used for a variety of analyses; the 512 women used in the present analysis are derived from this dataset^29^.

**Table 1:** Distributions of demographic data, tumor characteristics, treatment, and recurrence status by MRI use, among the women (age ≤ 50) diagnosed 2006–2013. Student’s *t*-tests were used for continuous variables and Pearson’s Chi-squared tests for categorical variables.

	MRI (330)	NO MRI (182)	*P*-value
Age	43.4 (5.0)	43.6 (5.2)	0.62
Race/ethnicity *N* (%)			0.65
Non-Hispanic white 379	244 (73.9%)	135 (74.2%)	
Non-Hispanic black 70	45 (13.6%)	25 (13.7%)	
Hispanic 30	18 (5.5%)	12 (6.6%)	
Asian 16	13 (3.9%)	3 (1.6%)	
Unknown 17	10 (3.0%)	7 (3.8%)	
Tumor size (cm)	1.64 (1.2)	1.80 (1.3)	0.18
Grade *N* (%)			0.79
Grade 1 117	73 (22.1%)	44 (24.2%)	
Grade 2 212	140 (42.4%)	72 (39.6%)	
Grade 3 183	117 (35.5%)	66 (36.3%)	
Histology *N* (%)			0.91
IDC 379	246 (74.5%)	133 (73.1%)	
DCIS 110	69 (20.9%)	41 (22.5%)	
ILC 23	15 (4.5%)	8 (4.4%)	
Nodal status *N* (%)			0.55
Positive 145	97 (29.4%)	48 (26.4%)	
Negative 296	185 (56.1%)	111 (61.0%)	
Unknown 71	48 (14.5%)	23 (12.6%)	
Tumor stage *N* (%)			0.80
Stage 0 110	69 (24.6%)	41 (26.1%)	
Stage I 220	139 (49.5%)	81 (51.6%)	
Stage II 61	40 (14.2%)	21 (13.4%)	
Stage III 47	33 (11.7%)	66 (8.9%)	
ER			0.98
Positive 415	267 (80.9%)	148 (81.3%)	
Negative 89	58 (17.6%)	31 (17.0%)	
Unknown 8	5 (1.5%)	3 (1.6%)	
PR			0.96
Positive 399	256 (77.6%)	143 (78.6%)	
Negative 107	70 (21.2%)	37 (20.3%)	
Unknown 6	4 (1.2%)	2 (1.1%)	
HER2			0.54
Positive 49	31 (9.4%)	18 (9.9%)	
Negative 355	234 (70.9%)	121 (66.5%)	
Unknown 108	65 (19.7%)	43 (23.6%)	
Systemic treatment *N* (%)	294 (89.1%)	160 (87.9%)	0.69
Median diagnosis date	10/2008	08/2007	
Follow-up length (years)	5.8 (2.6)	6.4 (2.6)	0.004
Re-excision	29 (8.8%)	21 (11.5%)	0.32
Local recurrence *N* (%)	26 (7.9%)	15 (8.2%)	0.88
Distant recurrence *N* (%)	21 (6.4%)	12 (6.6%)	0.92

### Overall population characteristics

Of 512 women aged ≤50, 330 (64.5%) underwent breast MRI and 182 (35.5%) did not. Since the increasing use of MRI has been documented, particularly for younger women^30,31^, we assessed whether, in this age group of women ≤50 years, there was an association between MRI use and age. Using a logistic regression equation with MRI as the output variable and age as the input variable, we found no association between age and MRI (coefficient = 0.014 and *p* = 0.40). Table 1 shows the demographic, tumor, and treatment characteristics of each group. The two groups were well-balanced, with similar age and race/ethnicity distribution; and no significant differences in tumor size, histology, grade, nodal, or hormone receptor status (Table 1). Mean age at diagnosis was similar for women who received MRI (43.4, SD = 5.0) and those who did not (43.6, SD = 5.2, *p* = 0.62). Among women who underwent MRI, 73.9% were of European descent, 13.6% were African-American, 5.5% were Hispanic, and 3.9% were of Asian origin. Racial/ethnic distribution was very similar in the no-MRI group (Table 1).

Tumor characteristics were also well-balanced, with no significant difference in tumor size (1.64 vs. 1.80, *p* = 0.18), or in the distribution of tumor grade (*p* = 0.79) or histology (*p* = 0.91). Nodal positivity was observed in 29.4% of women in the MRI group and 26.4% in the no-MRI group (*p* = 0.55). The majority of women had ER or PR positive tumors. Similar proportions of women received systemic therapy. A somewhat larger fraction of women in the MRI group had undergone mammography in the years preceding diagnosis (53.3 vs. 49.5%), whereas the average number of prior mammograms was about the same (5.1 SD = 4.3 in both groups). The average follow-up time after diagnosis was 5.8 years (SD = 2.6) for women with MRI and 6.4 years (SD = 2.6) for women without MRI (*p* = 0.004). Re-excision rates following first tumor resection were essentially the same, regardless of MRI use (8.8 vs. 11.5%, *p* = 0.32).

### Cancer outcomes

**Fig. 1:**
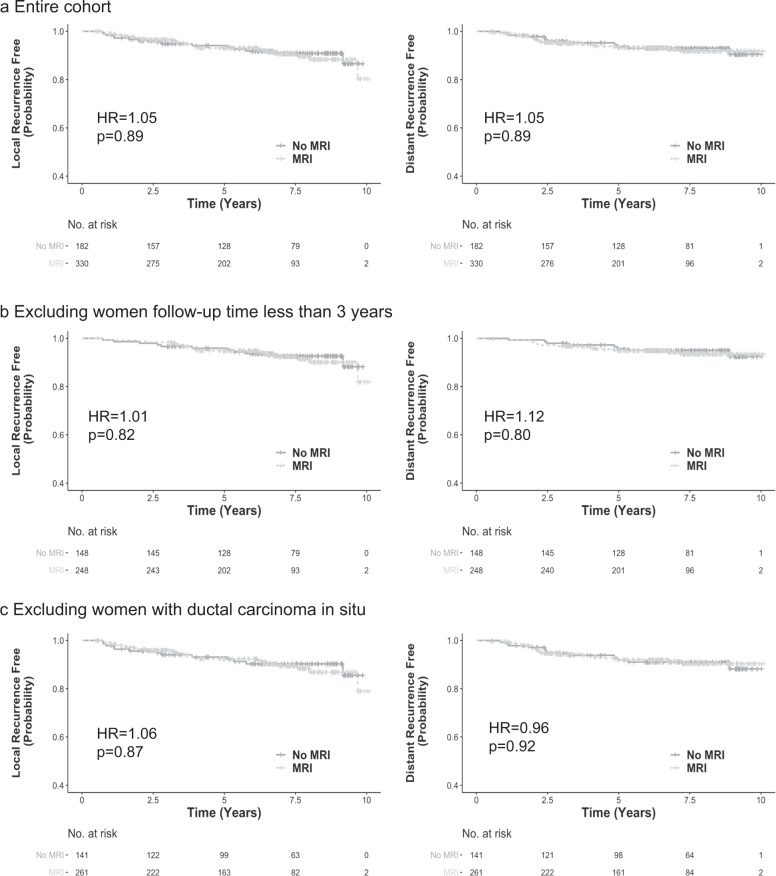
Kaplan–Meier local and distant recurrence-free survival curves for magnetic resonance imaging (MRI) versus no MRI. **a** Entire cohort. **b** Sensitivity analysis excluding women with follow-up time <3 years. **c** Sensitivity analysis excluding women with Ductal carcinoma in situ. *P*-values were calculated using log-rank test.

The frequency of local and distant recurrence is shown in Table 1. After an average of 5.8 years’ follow-up, local recurrence was observed in 26 (7.9%) women in the MRI group and 15 (8.2%) women in the no-MRI group; distant recurrence occurred in 21 (6.4%) women in the MRI group and 12 (6.6%) women in the no-MRI group. The cumulative incidence of local recurrence was not significantly lower in the MRI group, compared to the no-MRI group (7.9 vs. 8.2%, *p* = 0.88) (Table 1). Similarly, no significant difference was identified between the MRI group and the no-MRI group in the rate of distant recurrence (6.4 vs. 6.6%, *p* = 0.92) (Table 1). In univariable analyses including all 512 women, MRI use was not associated with local recurrence-free survival in a Cox regression model (HR = 1.05; 95% CI: 0.55–1.98; *p* = 0.89) (Table 2). Similarly, distant recurrence-free survival was not associated with MRI use either (HR = 1.05; 95% CI: 0.52–2.14; *p* = 0.89) (Table 2). The Kaplan–Meier local and distant recurrence-free survival curves for women who underwent MRI versus those who did not are shown in Fig. 1a.

**Table 2:** Univariable Cox regression model to test the association between MRI use and local/distant recurrence-free survival, among the women (age ≤ 50) diagnosed 2006–2013.

	Local recurrence		Distant recurrence	
	HR (95% CI)	*P*-value	HR (95% CI)	*P*-value
MRI use	1.05 (0.55, 1.98)	0.89	1.05 (0.52, 2.14)	0.89
Exclude follow-up time <3 years	1.01 (0.52, 2.30)	0.82	1.12 (0.47, 2.67)	0.80
Exclude women with DCIS	1.06 (0.54, 2.08)	0.87	0.96 (0.47, 1.97)	0.92

Sensitivity analyses were performed by excluding women with follow-up time under 3 years, or DCIS.

We then performed multivariable analyses, with adjustment of covariates, to evaluate the association between MRI and local recurrence-free survival. In the multivariable model, we adjusted for age, race/ethnicity, tumor size, tumor grade, lymph node status, ER status, HER2 status, P53 status, and systemic therapy status (including chemotherapy and endocrine therapy). The association with local recurrence-free survival remained non-significant for the main effect of MRI use (aHR = 1.03; 95% CI: 0.53–1.99; *p* = 0.94) (Table 3). No other parameters were significantly associated with the hazard of local recurrence. Similarly, association between MRI and distant recurrence-free survival remained non-significant following adjustment of covariates (aHR = 0.89; 95% CI: 0.43–1.84; *p* = 0.74). In the multivariable model, tumor size, ER status, and nodal positivity were significantly associated with distant recurrence-free survival.

**Table 3:** Multivariable cox regression model to test the association between MRI use and local/distant recurrence-free survival, among the women (age ≤ 50) diagnosed in 2006–2013.

	Local recurrence		Distant recurrence	
	HR (95% CI)	*P*-value	HR (95% CI)	*P*-value
MRI (reference, none *N* = 182)				
MRI performed *N* = 330	1.03 (0.53, 1.99)	0.94	0.89 (0.43, 1.84)	0.74
Age	1 (0.94, 1.06)	0.94	0.98 (0.92, 1.04)	0.56
Race (reference, non-Hispanic whites *N* = 379)				
Non-Hispanic black *N* = 70	1.47 (0.64, 3.40)	0.37	1.6 (0.71, 3.6)	0.25
Hispanic *N* = 30	1.46 (0.42, 5.00)	0.55	1.47 (0.33, 6.6)	0.62
Asian *N* = 16	0.79 (0.10, 6.05)	0.82	2.17 (0.47, 10.11)	0.32
Tumor size	1.16 (0.91, 1.48)	0.23	1.47 (1.19, 1.82)	<0.001
Grade (reference, Grade 1 *N* = 117)				
Grade 2 *N* = 212	1.98 (0.63, 6.16)	0.24	4.79 (0.62, 37.33)	0.13
Grade 3 *N* = 183	2.00 (0.54, 7.36)	0.30	3.11 (0.34, 28.73)	0.32
Nodal status (reference, Negative *N* = 296)				
Positive *N* = 145	1.36 (0.68, 2.73)	0.38	2.14 (1.01, 4.53)	0.05
Histology (reference, IDC *N* = 379)				
DCIS *N* = 110	0.57 (0.11, 2.82)	0.49	1.45 (0.18, 11.77)	0.73
ILC *N* = 23	0.59 (0.07, 4.80)	0.62		
ER (reference, Negative *N* = 89)				
Positive *N* = 415	0.69 (0.28, 1.71)	0.43	0.3 (0.11, 0.85)	0.02
HER2 (reference, Negative *N* = 355)				
Positive *N* = 49	1.34 (0.53, 3.40)	0.54	0.74 (0.22, 2.52)	0.63
P53 (reference, Negative *N* = 246)				
Positive *N* = 89	1.53 (0.70, 3.37)	0.29	0.91 (0.38, 2.2)	0.84
Systemic therapy (reference, none *N* = 58)				
Performed *N* = 454	0.63 (0.19, 2.12)	0.46	0.48 (0.1, 2.23)	0.35

Adjusted for age, race/ethnicity, tumor size, tumor grade, lymph node status, ER status, HER2 status, P53 status, and systemic therapy status (including chemotherapy and endocrine therapy). Number of local recurrences among the 512 women was 41 (8.0%). Number of distant recurrences among the 512 women was 33 (6.4%).

Competing risk factors for the recurrences were examined as well. There was no substantial change in the associations when allowing for competing risk of death from any cause in univariable models for local recurrence (HR = 1.04; 95% CI: 0.55–1.96; *p* = 0.91) and distant recurrence (HR = 1.05; 95% CI: 0.52–2.11; *p* = 0.90). In the multivariable models adjusted for covariates, no substantial change was found when allowing competing risks of distant recurrence for local recurrence (aHR = 1.04; 95% CI: 0.51–2.13; *p* = 0.91) (Table 4a). Allowing competing risks of death from any cause did not make substantial change for local recurrence (aHR = 1.04; 95% CI: 0.50–2.04; *p* = 0.97) or distant recurrence (aHR = 0.88; 95% CI: 0.43–1.83; *p* = 0.74) (Table 4b). When allowing competing risks of distant recurrence and death from any cause for local recurrence, the association remained non-significant (HR = 1.04; 95% CI: 0.53–2.12; *p* = 0.92) (Table 4b).

**Table 4:** Competing risk analyses and sensitivity analyses to test the association between MRI use and local/distant recurrence-free survival, among the women (age ≤ 50) diagnosed in 2006–2013.

	Local recurrence		Distant recurrence	
	HR (95% CI)	*P*-value	HR (95% CI)	*P*-value
a. *Competing risks*^a^				
No Competing risk	1.03 (0.53, 1.99)	0.94	0.89 (0.43, 1.84)	0.74
DR as competing risk	1.04 (0.51, 2.13)	0.91	NA	NA
Death as competing risk	1.01 (0.50, 2.04)	0.97	0.88 (0.43, 1.83)	0.74
DR & death as competing risks	1.04 (0.51, 2.12)	0.92	NA	NA
b. *Sensitivity analyses*^a^				
Exclude follow-up time <3 years				
*N* = 396	1.16 (0.53, 2.55)	0.70	0.87 (0.36, 2.14)	0.77
Exclude women with DCIS				
*N* = 402	1.08 (0.53, 2.22)	0.82	0.85 (0.40, 1.78)	0.66
Women ≤ 40 years old				
*N* = 119	1.82 (0.43, 7.76)	0.42	0.93 (0.26, 3.34)	0.91

a: Competing risk models^a^ showing association between MRI use and local/distant recurrence-free survival, allowing distant recurrence to compete with local recurrence, and death to compete with both recurrence types. b: Sensitivity analyses^a^ excluding women with follow-up time under 3 years, or DCIS, or women aged 40 or less.

^a^Cox regression models adjusted for age, race/ethnicity, tumor size, tumor grade, lymph node status, ER status, HER2 status, P53 status, systemic therapy status (including chemotherapy and endocrine therapy).

Sensitivity analyses were further performed to examine the associations between MRI use and local/distant recurrences in both univariable and multivariable models. In sensitivity analyses that excluded women with follow-up time <3 years, the associations remain non-significant for local recurrence (HR = 1.01; 95% CI: 0.52–2.30; *p* = 0.82) and distant recurrence (HR = 1.12; 95% CI: 0.47–2.67; *p* = 0.80) in univariable models (Table 2). We then removed 110 women with a DCIS diagnosis and repeated the analyses. The association with local recurrence remained non-significant (HR = 1.06; 95% CI: 0.54, 2.08; *p* = 0.87), as did that with distant recurrence (HR = 0.96; 95% CI: 0.47–1.97; *p* = 0.92). Kaplan–Meier local and distant recurrence-free survival curves for the MRI group versus no-MRI group are shown in Fig. 1b and c, respectively. We then performed these sensitivity analyses adjusting for the same covariates as in the main model. The associations between MRI and local/distant recurrence-free survival also remain non-significant (Table 4b).

To clarify the potential benefit of preoperative breast MRI on the youngest women, we further evaluated cancer outcomes following preoperative MRI use among women aged 40 years or less in multivariable models. While 119 women were included in this analysis, their chance of receiving MRI was comparable (84 MRI vs. 44 no MRI, 1.9-fold) to the full cohort higher (330 MRI vs. 182 no MRI, 1.8-fold). There were no significant differences in age (36.2 vs. 35.6, *p* = 0.39), follow-up length, race distribution, tumor size, histology, grade, nodal or hormone receptor status, or systemic therapy between the MRI and no MRI group. When adjusted for important tumor and treatment parameters, we found no difference in the hazard of local (aHR = 1.82; 95% CI: 0.43–7.76; *p* = 0.42) or distant recurrence (aHR = 0.93; 95% CI: 0.26–3.34; *p* = 0.91) in women who underwent preoperative MRI and those who did not, suggesting that women aged 40 years or less did not fare better with MRI-guided surgical treatment than their counterparts who were treated without MRI (Table 4b).

## Discussion

The potential benefit of preoperative breast MRI for evaluation of disease extent in newly diagnosed breast cancer patients can be assessed at two levels: short-term surgical outcomes (appropriate modification of surgical plans and reduced positive margin rates) and long-term outcomes—does MRI guidance of the surgical plan improve local control? The short-term endpoint has been addressed in other studies, with mixed results^6,16,30,32,33^. In the present study, we have addressed the second, arguably more meaningful outcome, namely locoregional recurrence risk specifically in younger women. The relevance of a local control outcome to MRI utilization is widely acknowledged in the prior literature, where the use of this diagnostic test has been analyzed with regard to cancer recurrence outcomes, both locoregional and distant^2,34–37^. The results of these are summarized in Table 5; they show, almost uniformly, no improvement in locoregional control when surgical resection is informed by MRI evaluation of disease extent.

**Table 5:** Local recurrence events in studies of preoperative MRI use and breast cancer outcomes^a^.

	Number of events	Sample size	Median follow-up (month)^b^	IBTR (MRI vs. no MRI)	HR (95% CI) or *P* for event rate
Vapiwala et al.^22^^c^	49	755	166	8% vs. 8%	0.98 (0.52, 1.87)
Hill et al.^19^	78	1396	86 (MRI) and 77 (no MRI)	8 vs. 4%	
Choi et al.^21^	66	1598	Over 60	4.0 vs. 4.3%	0.95 (0.58, 1.54)
Gervais et al.^37^	17^d^	470	85 (MRI) and 106 (no MRI)	1.6 vs. 4.2%	2.0 (0.45, 9.00)
Yi et al.^15^	27	936	74	1.7 vs. 4.1%	0.3 (0.1, 0.9)
Pilewskie et al.^20^ DCIS only	184	2321	59	8.5 vs. 7.2%	1.36 (0.78, 2.39)
Sung et al.^16^	22	348	96	5 vs. 9%	*P* = 0.33
Miller et al.^44^	9	414	29 (MRI) and 45 (no MRI)		*P* = 0.13
Hwang et al.^17^	11	472	54	1.8 vs. 2.5%	1.7 (0.2, 11.8)
Turnbull et al.^9^^e^	52	1569	24	1.5 vs. 1.5%	Not reported
Fischer et al.^14^^f^	10	346	41	1.2 vs. 6.5%	*P* = 0.001

^a^The meta-analysis of Houssami et al. is not included in the table, since the component studies are included.

^b^Follow-up is presented separately by MRI status when so reported by authors.

^c^Updated analysis of Solin et al.^18^.

^d^Number of events estimated from reported rates at 8 years.

^e^Reported in Houssami meta-analysis.

^f^Outcomes not adjusted for tumor or therapy characteristics.

Almost all studies have been retrospective (with the exception of the prospective randomized COMICE trial^9^) with a propensity toward the MRI group being younger than the conventional imaging group^6,11,17,18,21–23^. The increasing use of preoperative breast MRI from 2005 onward has been documented in health claims data for women under 65, with the odds of receiving MRI being highest in the youngest women^30,31,38^. This consistent trend betrays the perception that younger women (with denser breast tissue and lower sensitivity of mammography) are more deserving of MRI, and can expect greater benefit^39^. Given the more firmly held expectations regarding the benefits of breast MRI in younger breast cancer patients, this age group deserves further attention to confirm the benefits (or lack thereof) of MRI in the surgical setting. However, no previous study has purposefully examined this group. We evaluated cancer outcomes following preoperative MRI use with a focus on women aged 50 years or less, diagnosed in 2006 or later. We found no difference in the hazard of local or distant recurrence in women who underwent preoperative MRI and those who did not. This was true of crude recurrence rates; of hazard ratios resulting from univariate analysis; and of hazard ratios that were adjusted for important tumor and treatment parameters. These findings persist in an analysis of women aged 40 years or less, where the fraction of patients undergoing MRI was similar to the whole study population (65%). Thus, our results suggest, the biology of breast cancer in younger women is no more amenable to the possible advantages of better anatomic resection achieved with better imaging than in women of all ages that were included in previous studies. We found that women aged 50 or less at diagnosis did not fare better with MRI-guided surgical treatment than their counterparts who were treated without MRI.

Our results are in agreement with previous studies, summarized in Table 5. The individual-level meta-analysis of three retrospective studies and the COMICE trial is not detailed in Table 5, since its component studies are described. The meta-analysis was based on a pooled dataset of 3180 women and a total of 64 local recurrences in women with a median follow-up time of 3 years. The meta-analysis, and the individual studies^6,16,17,40^. showed no benefit for MRI use in the prevention of subsequent breast cancer events. Only two of the ten studies published so far have reported a positive effect of MRI use on cancer outcomes, but both described very few recurrence events. Of note, the report by Fischer et al.^14^ did not adjust for key prognostic factors (tumor characteristics, adjuvant systemic therapy, etc.); interpretation also rendered difficulties since they reported a series of 346 women who experienced 10 local recurrence events. Few previous studies have reported on distant recurrences with reference to preoperative MRI use. Those that have, found no significant association between preoperative MRI use and distant recurrence-free survival^15,16,18,21^. Our results are similar both in the present analysis of women aged ≤50 and those 50 years or older (data not shown). This is not surprising, since presumably any effect of better local therapy through better local disease evaluation would have a bigger effect on local than on distant control.

Strengths of our study include the fact that we focused on the period after 2006, when MRI use had stabilized at our institution, thus reducing between-group differences in follow-up time (a feature of most previous studies^9,14–18^) and in surgical and pathological practice (e.g., use of cavity-shave margins). Our study population was restricted to those aged ≤50 years, MRI and no-MRI groups were well-balanced in terms of age and race/ethnicity distribution. There were no significant differences in tumor size, histology, grade, nodal or hormone receptor status. In competing risk models, distant recurrence and death were allowed to compete for local recurrence, and death was allowed to compete for distant recurrence. Again, we found hazard ratios that remained close to unity for both local and distant recurrence, with no significant differences between MRI and no-MRI groups. Unlike previous studies, we used sensitivity analyses that excluded women with DCIS (*N* = 110), and those who had follow-up time of <3 years (*N* = 116), and again found no suggestion of a decrease in recurrences in women who received preoperative MRI, after adjustment for important covariates. Our dataset was rigorously developed using a combination of natural language processing and manual chart review^25–27^. We examined breast cancer outcomes in 512 young women aged ≤50 years, taken from a larger cohort encompassing all ages. In our study population, the use of adjuvant therapeutic modalities in MRI recipients was driven by differences in patient and tumor characteristics, such as tumor size and nodal positivity, as in previous studies^6,19,21^. As expected in a younger population, we observed a higher rate of local recurrence (8.0%) than in studies where age was not restricted by study design. In the entire cohort of women treated at our institution over the same time-frame local recurrence rate is 5.1%, and also uninfluenced by MRI use (*p* = 0.03, data not shown).

The weakness of our study is similar to previously published studies on cancer outcomes related to preoperative breast MRI, in that it is retrospective. Like other retrospective studies, our MRI group had shorter follow-up (by about 10 months) than the conventional imaging group; three previous authors have reported a somewhat shorter follow-up period in the MRI group [refs] whereas others have not specified follow-up period specifically for each group (see Table 5). All other differences were minimized by the focus on women aged 50 or younger, diagnosed with primary breast cancer in 2006 or later, and by performing a sensitivity analysis that excluded women with DCIS. We did not specifically examine breast density in our study. But an analysis by Elder et al. of 683 newly diagnosed patients undergoing MRI, of whom two-thirds had dense breasts on mammography, sheds light on the relationship between mammographic density, MRI findings, and local control^41^. They reported that MRI abnormalities were more common among women with high-density breasts (ipsilateral 41.8%, contralateral 24.9%) than women with low-density breasts (ipsilateral 30.7%, contralateral 13.8%), but additional cancer was not diagnosed more frequently^41^. Overall, with a median follow-up of 89 months, local recurrence was not different in women with dense versus non-dense breasts ^41^.

The potential benefits of preoperative MRI remain to be evaluated in prospective trials. The on-going Alliance AO11104 trial (NCT01805076) will resolve some of these issues for women with triple-negative, ER-poor, or HER2-positive breast cancer. However, the trial will not apply to patients with hormone receptor-positive tumors (the majority of breast cancer patients), and is powered to detect an absolute 8% improvement in local recurrence, with a hazard ratio of 0.19, which is a challenging goal. The COMICE trial^9^ will provide more generalizable data but has been criticized in the United States with regard to the quality of MRI^40^ and therefore (regrettably) data on long-term outcomes may also be questioned. Our results illustrate that at our institution (and likely in others) preoperative MRI is still considered valuable in younger women; but here too, as in studies that did not address the effect of age, we see no evidence for improved outcomes. Despite the uniformity of the negative data on preoperative MRI use, it continues to be advocated. As surveillance MRI becomes more widely used, as seems likely, the specific decision regarding preoperative use may become less important; on the other hand, as adjuvant therapy improves, the anatomic delineation of disease may also become less important. This tenet is supported by the fact that multivariable models examining the contribution of MRI use to recurrence outcomes continue to show the independent effect of tumor biology and therapy, regardless of MRI use. Therefore, particularly in resource-limited environments, it is important for practitioners to recognize the present lack of evidence regarding the benefits of preoperative breast MRI vis a vis cancer outcomes, and to counsel patients accordingly.

## Methods

### Clinical population

Women presenting to the Lynn Sage Breast Center of Northwestern Medicine for surgical therapy of a recently diagnosed non-metastatic breast cancer (invasive or DCIS) were included. Eligible subjects provided informed consent for the use of their clinical data for research studies, and the study has been approved by the IRB board at Northwestern University (IRB number STU00200923-MOD0006). They were aged 50 years or less at diagnosis, and their final surgical procedure was breast conservation. Those undergoing neoadjuvant systemic therapy were excluded. A minimum follow-up period of 6 months was required. Patients were then categorized into those who did or did not receive breast MRI as part of their extent of disease evaluation for surgical planning. Breast MRI utilization was determined either at an outside institution (if the diagnosis had been made elsewhere) or by the primary care physician at the recommendation of the radiologist, or by the surgical team after discussion with the imaging physician and after the presentation of risks and benefits to the patient. The risk and benefit discussion included a description of greater sensitivity of MRI, a higher false-positive rate, the possibility of additional biopsies, and a lack of evidence regarding improved outcomes. The radiologist’s recommendation for MRI use included consideration of breast density. Reasons for patient acceptance of MRI were not recorded, but when it was declined, it was usually based on claustrophobia, a fear of additional biopsies, or concerns about out of pocket expenses. Breast-conserving surgery was performed in standard fashion, with re-excision performed for positive margins, or when more than one margin within 1 mm. All patients were referred to radiation oncology for consultation. Patients who resided some distance away so that travel to Northwestern on a daily basis was not feasible sought radiotherapy services closer to home. The delivery of RT elsewhere was documented in the follow-up records when they returned to Northwestern for surveillance visits. Patients without clear documentation of RT use were excluded from analysis, since radiotherapy is a required component of breast-conserving therapy in this age group. Systemic therapy decisions were made in consultation with medical oncology practitioners. Follow-up data were obtained from surveillance visits to surgical, medical, or radiation oncology offices at Northwestern, and was supplemented by Tumor Registry records.

### Statistical analyses

The MRI and no-MRI groups were the main focus of analysis. Descriptive statistics of continuous variables were summarized as mean and standard deviation (SD); between-group differences were evaluated with Student’s *t*-tests. For discrete variables, the number and percentage in each category were compared, using Pearson’s Chi-squared tests to test between-group differences. Using Cox regression, hazard ratios (HR) with 95% confidence intervals (CI) were computed for the MRI group relative to the no-MRI group. Kaplan–Meier survival curves were generated for time to local recurrence and time to distant recurrence. Furthermore, *p*-values were computed using the log-rank test to test between-group differences. The multivariable model adjusted for confounding factors including age, race/ethnicity, tumor size and grade; lymph nodes positivity, ER, HER2, and P53 status; use of radiation, and systemic therapy. Due to the high correlation between ER status and PR status, only ER status was included in the model. Sensitivity analyses were performed by excluding women with follow-up time <3 years, or those with DCIS.

Local recurrence is not independent of distant recurrence^42^. Distant recurrence and death are competing events for local recurrence for each individual patient, thus estimating time to local recurrences turns into estimating competing risks^42^. To address competing risks, models were fitted to assess the effect of loss to follow-up as a result of distant recurrence or death on local recurrence estimates, and loss to follow-up as a result of death on distant recurrence estimates. In the competing risk models, when using the distant recurrence to compete with local recurrence, women who had a distant recurrence without or before local recurrence are no longer considered for local recurrence at the time of distant recurrence. The proportional hazards model described by Fine and Gray was used for this competing risk analysis^43^.

### Reporting summary

Further information on research design is available in the Nature Research Reporting Summary linked to this article.

## Supplementary information

Supplemental Data

Reporting Summary Checklist FLAT

## Data Availability

The data generated and analyzed during this study are described in the following data record: 10.6084/m9.figshare.12777140^29^. The data supporting the related study and underlying all figures and tables in the related manuscript are collected in the file “df_MRI_de-identified.csv”. This file is not publicly available due to data privacy policies regarding participant consent: patients did not provide consent to share their data publicly. However, the data can be provided upon request to the corresponding author.

## Code availability

All codes necessary to re-generate the results are publicly available at https://github.com/zexian/MRI_codes.
